# Discovery of new candidate genes for rheumatoid arthritis through integration of genetic association data with expression pathway analysis

**DOI:** 10.1186/s13075-017-1220-5

**Published:** 2017-02-02

**Authors:** Klementy Shchetynsky, Lina-Marcella Diaz-Gallo, Lasse Folkersen, Aase Haj Hensvold, Anca Irinel Catrina, Louise Berg, Lars Klareskog, Leonid Padyukov

**Affiliations:** Rheumatology Unit, Department of Medicine Centre of Molecular Medicine, CMM:L8:04, Karolinska Institutet/Karolinska University Hospital Solna, 171 61 Stockholm, Sweden

**Keywords:** Rheumatoid arthritis, Molecular genetics, Immunology, Genome-wide, RNA-seq

## Abstract

**Background:**

Here we integrate verified signals from previous genetic association studies with gene expression and pathway analysis for discovery of new candidate genes and signaling networks, relevant for rheumatoid arthritis (RA).

**Method:**

RNA-sequencing-(RNA-seq)-based expression analysis of 377 genes from previously verified RA-associated loci was performed in blood cells from 5 newly diagnosed, non-treated patients with RA, 7 patients with treated RA and 12 healthy controls. Differentially expressed genes sharing a similar expression pattern in treated and untreated RA sub-groups were selected for pathway analysis. A set of “connector” genes derived from pathway analysis was tested for differential expression in the initial discovery cohort and validated in blood cells from 73 patients with RA and in 35 healthy controls.

**Results:**

There were 11 qualifying genes selected for pathway analysis and these were grouped into two evidence-based functional networks, containing 29 and 27 additional connector molecules. The expression of genes, corresponding to connector molecules was then tested in the initial RNA-seq data. Differences in the expression of *ERBB2*, *TP53* and *THOP1* were similar in both treated and non-treated patients with RA and an additional nine genes were differentially expressed in at least one group of patients compared to healthy controls. The *ERBB2*, *TP53. THOP1* expression profile was successfully replicated in RNA-seq data from peripheral blood mononuclear cells from healthy controls and non-treated patients with RA, in an independent collection of samples.

**Conclusion:**

Integration of RNA-seq data with findings from association studies, and consequent pathway analysis implicate new candidate genes, *ERBB2*, *TP53* and *THOP1* in the pathogenesis of RA.

**Electronic supplementary material:**

The online version of this article (doi:10.1186/s13075-017-1220-5) contains supplementary material, which is available to authorized users.

## Background

Implementation of next-generation sequencing (NGS) to analyse RNA expression has revolutionized our capacity to approach different cell functions. However, the precise functional interpretation of whole-transcriptome expression data is still challenging [1]. Currently, there are multiple approaches to analysing the output of RNA-sequencing (RNA-seq), but there is little consensus on the algorithms used to process and interpret the data. Integration of multiple RNA-seq data sets with extensive amounts of data from genome-wide association studies (GWAS) and protein interaction databases may improve the interpretation of data and contribute to the discovery of new molecular targets that are supported by experimental evidence on multiple levels [2].

Here we attempted to incorporate previous knowledge from genome-wide association studies and pathway analyses into the framework of an RNA-seq-based study of differential gene expression in rheumatoid arthritis (RA). RA is a common autoimmune disease, manifesting as chronic inflammation of the joints, and characterized by a significant genetic contribution [3–5] and gender bias (1.8–3.6 female-to-male ratio) [6, 7].

Refinement and meta-analysis of large-scale GWAS have found associations between risk of RA and more than a hundred variants in non-human leukocyte antigen (HLA) loci [8–11]. A comprehensive review by Okada et al. summarises a multitude of verified genetic variants playing a role in RA susceptibility, suggesting 377 genes for prospective study, based on their proximity to verified RA-associated single nucleotide polymorphisms (SNPs) [12]. Multiple previous studies suggest enrichment for cis-acting variants, located in close proximity to coding genes [13].

Focusing on previously validated genetic variants associated with RA (with genome-wide significant association), we used RNA-seq to address the expression of the genes proximal to these SNPs, in whole-blood samples from patients with RA and from healthy individuals. We differentiated between treated and non-treated patients with (early) RA to identify common expression signals in these two groups, and controlled for the effects of treatment when comparing them to healthy controls. We applied pathway analysis to differentially expressed genes from RNA-seq data, which correspond to previously validated genetic variants associated with RA, with the goal of identifying new functionally meaningful candidate genes and gene networks playing a role in the disease. A new gene set derived from interconnections in pathway analysis, was analyzed for differential expression and validated in a larger independent collection of samples, sharing a similar structure with our discovery cohort.

## Methods

### Cohorts of patients and controls

We studied 12 female patients from Karolinska University Hospital, Solna, Sweden, who had RA that corresponded to the American College of Rheumatology (ACR) 1987 criteria and the European League Against Rheumatism (EULAR)/ACR 2010 criteria for RA, according to assessment by trained rheumatologists. Of the 12 patients with RA, 5 were non-treated and had not previously used anti-rheumatic drugs (patients with early RA with symptom duration less than 1 year), and 7 of the patients were receiving anti-rheumatic treatment (either methotrexate or biological agents). Patients with early RA, not yet receiving anti-rheumatic treatment, could still receive non-steroid anti-inflammatory drugs (NSAIDs). The study also included 12 matched healthy female individuals as a control group. An additional non-overlapping validation cohort (COMBINE) consisted of 46 non-treated female patients with RA, 27 female patients with RA treated with methotrexate and 34 healthy female controls. Additional clinical information for the studied groups, including age range, disease activity score in 28 joints (DAS28) and anti-citrullinated peptide antibody (ACPA) status is presented in Additional file 1: Table S1.

### RNA extraction and sequencing

Whole blood from all patients and healthy controls was collected in PAXgene Blood RNA Tubes according to the manufacturer’s protocol and saved at -20 °C. RNA was extracted using PAXgene Blood miRNA kit (PreAnalytiX, Switzerland). Sample quality was assessed using Agilent Technologies Bioanalyzer with Agilent RNA 6000 Nano Kit (Agilent Technologies, Sweden). RNA-seq was based on the Illumina HiSeq 2000 platform and TruSeq library construction, with 20-m pair-end reads per sample (Illumina, CA, USA). For the RNA-seq data from the initial discovery cohort and the COMBINE validation cohort, TopHat-Cufflinks 2.0 software package was used for RNA-seq data alignment and analysis, as described previously [14], against an hg19 USCS human genome reference. Cufflinks 2.0 was used for abundance estimation and quantification of transcripts. These data have been deposited in the NCBI Gene Expression Omnibus repository [GEO:GSE90081] [15]. The PBMC samples for the COMBINE validation cohort were collected as citrated blood and isolated using Ficoll-Paque (GE Healthcare, Sweden). A detailed description of the validation cohort has been published previously [16].

### Pathway analysis

RA-associated loci were chosen based on the selection reported previously by Okada et al. [17], and corresponding proximal genes were defined as being either 2 kbp away or in linkage disequilibrium of *r*2 > 0.50 with any of the 100 verified non-HLA RA risk loci from this study. The workflow is presented schematically in Additional file 2: Figure S1. The network construction was based on the Ingenuity Pathway Analysis (IPA) (QIAGEN, CA, USA) experimental evidence database (human, mouse and rat). The IPA score of 5 and above was used to assign a credible interaction network, requiring that the latter contains at least five of the input genes. The same input dataset was used for analysis with no restrictions on tissue type and a separate analysis, restricted to experimental data from immune primary cells and immune cell lines.

### Statistics

A significance cutoff of 0.05 was used for Cuffdiff results of comparison of either of the RA patient groups to controls, and followed by visualisation with R-based cummeRbund software [14]. The Cuffdiff approach to calculate differential gene expression is based on the beta negative binomial model to estimate the variance of the RNA-seq data by *t*-like statistics from fragments per kilobase of transcript per million mapped reads (FPKM) values [18].

The Cuffdiff-derived *p* values of genes with similarly directed fold changes from the separate analyses of treated and non-treated patients with RA were meta-analysed, using Fisher’s combined probability test. The results were adjusted for the number of tests performed, using Benjamini and Hochberg false discovery rate (FDR) correction with the adjusted *p* value threshold of 0.05 [19].

Quantitative expression data in the COMBINE validation cohort was analysed by the non-parametric Kruskal-Wallis H test. The threshold for significance was *p* = 0.05. For multiple tests, a false discovery rate threshold *q* value of 0.05 was used. Simple linear regression was used to assess the effect of gene expression on DAS28 score.

## Results

### Genes proximal to RA associated variants are differentially expressed

**Fig. 1:**
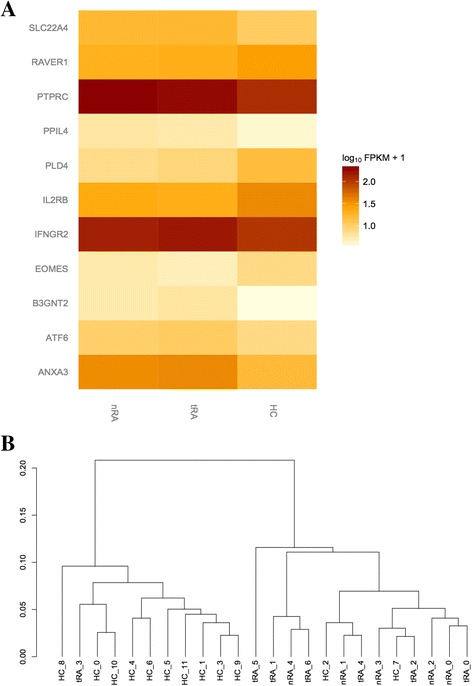
**a** Of the 377 genes associated with rheumatoid arthritis (RA) that were reported by Okada et al., 11 were uni-directionally differentially expressed in whole blood from both treated patients (*tRA*) and untreated patients (*nRA*) with RA, when compared to 12 healthy controls (*HC*). The *p* values from both analyses were combined by Fisher’s method. A significance threshold of *p* = 0.05 after correction for false discovery rate was applied. *FPKM* fragments per kilobase of transcript per million mapped reads. **b** Clustering on the 11-gene expression profile for grouping of individual samples using Jensen-Shannon distance (shown on axis Y)

Candidate genes were chosen based on proximity to validated genetic variants from previous GWAS and meta-analyses, summarized in the publication by Okada et al. as shown in Additional file 3: Data sheet [17]. Out of 377 genes from RA-associated loci, 22 genes were differentially expressed (DE) based on comparison of RNA-seq data between any of the two groups (with non-treated RA or treated RA) with controls after correction for multiple testing, as shown in (Additional file 4: Figure S2). However, the expression difference was significant only for 11 genes and was unidirectional in both non-treated and treated RA compared to healthy controls (Fig. 1a). As expected, clustering based on the expression pattern of these 11 genes resulted in a reasonably good separation of healthy controls and patients with RA, with group mismatches for only a single patient with RA and two control individuals (Fig. 1b). Predictably, there was no clear distinction between the groups with different treatment status.

### Pathway-based analysis of differentially expressed genes reveals new candidate genes for RA

**Fig. 2:**
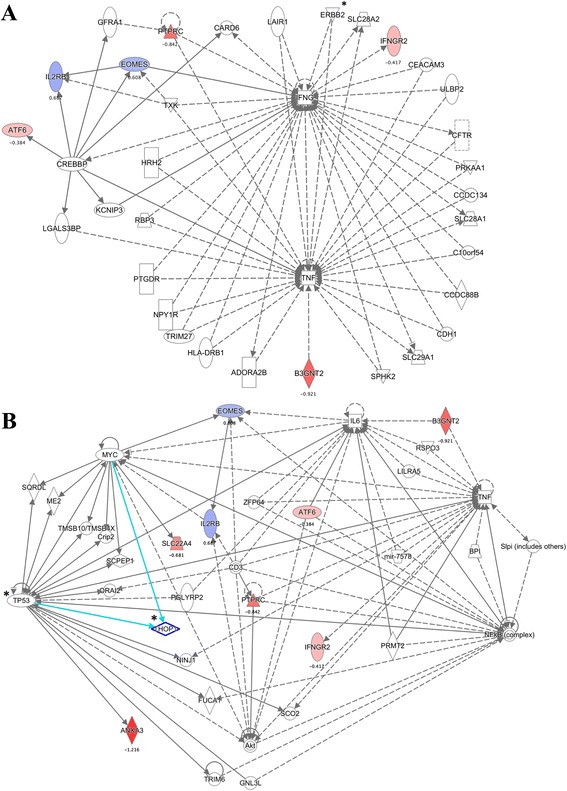
**a** A network derived from experimental data for immune cells containing corresponding input molecules for six genes, which were differentially expressed in whole blood RNA-sequencing (RNA-seq) data (Ingenuity Pathway Analysis (IPA)). **b** A network derived from experimental data with no tissue filter applied, containing corresponding input molecules for eight genes, which were differentially expressed in whole blood according to RNA-seq data. *Coloured shapes* represent genes previously associated with RA, which were also DE in RNA-seq data (input data) - expression log2 fold change is represented with colour intensity (*red* increased expression in patients with RA; *blue* decreased expression in patients with RA) and a corresponding value (RA patients versus healthy controls); *white shapes* represent interaction molecules; *solid lines* direct interaction evidence; *broken lines* indirect interaction evidence; *verified differentially expressed connector molecules, IPA

As an outcome of the pathway analysis, corresponding molecules for 6 out of 11 DE genes were interconnected in a reasonably sized functional molecular network, involving 29 additional interacting molecules (Fig. 2a), based on the database for immune cells and cell-lines within Ingenuity Pathway Analysis (IPA). These networks included genes functionally attributed to cellular development, cellular growth and proliferation, and haematological system development and function. An additional inclusive analysis was carried out without restrictions on tissue specificity (Fig. 2b). The resulting network contained corresponding molecules for 8 out of 11 DE genes and 27 additional “connectors”. TNF and molecules corresponding to 6 out of 11 input genes were present in both networks.

We used the set of connector molecules produced by pathway analysis to re-interrogate the initial RNA-seq data: 24 IPA-derived connector molecules had quantifiable expression values in our RNA-seq data, and 11 were DE (Additional file 1: Table S2). The meta-analysis approach was used for genes that were DE in a unilateral fashion in both RA groups, when compared to healthy controls. These new candidate genes were selected for further validation.

### Replication of expression of differentially expressed genes confirms three new candidates

To replicate our findings we employed analysis of RNA-seq data from the independent COMBINE validation cohort of 46 non-treated female patients with RA, 27 methotrexate-treated female patients with RA and 34 healthy female controls. On the discovery stage we noticed that *ERBB2* RNA expression in whole blood from both non-treated and treated patients with RA was significantly lower than in healthy controls (Kruskal-Wallis test *p* = 0.04).

**Fig. 3:**
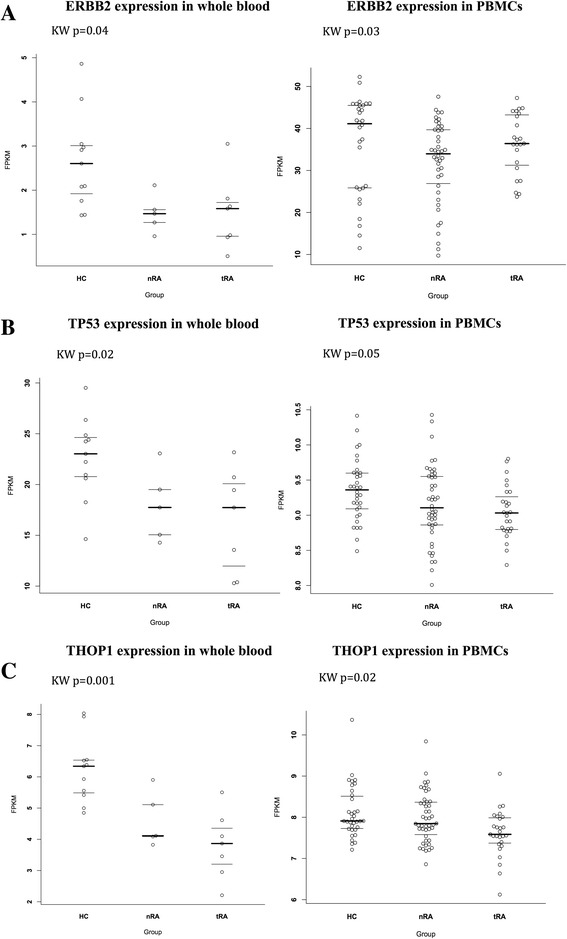
**a** *ERBB2* was significantly differentially expressed (DE) in whole blood from 5 non-treated patients with rheumatoid arthritis (*nRA*) and 12 healthy controls (HC) and for 7 treated patients with RA (*tRA*) versus 12 healthy controls (*p* = 0.04). The expression difference between nRA and HC was replicated in peripheral blood mononuclear cell (*PBMC*) samples from an independent cohort of 46 nRA, 27 methotrexate-treated patients with RA and 34 HC (*p* = 0.033). **b** *TP53* was differentially expressed in whole blood from 5 nRA and 7 tRA versus 12 HC (*p* = 0.02). There was a similar TP53 expression profile in PBMC samples from an independent cohort of 46 nRA, 27 methotrexate-treated patients with RA versus 34 HC (*p* = 0.05). **c** *THOP1* was differentially expressed in whole blood from 5 nRA and 7 tRA versus 12 healthy controls (p = 0.001); there was a significant difference in expression between 27 tRA and 34 HC in PBMC samples (*p* = 0.02). A *p* value of 0.05 was used as the significance threshold for the Kruskal-Wallis (*KW*) non-parametric test. *FPKM* fragments per kilobase of transcript per million mapped reads

A similar pattern was observed for *ERBB2* in the COMBINE validation cohort (Fig. 3a), with a significant difference in ERBB2 expression between healthy controls and non-treated patients (Kruskal-Wallis test *p* = 0.03). We also observed significantly lower *TP53* mRNA in non-treated and treated patients with RA compared to healthy controls in the discovery cohort (Kruskal-Wallis test *p* = 0.02) and subsequently demonstrated a similar expression profile in the COMBINE validation cohort (Kruskal-Wallis test *p* = 0.05) (Fig. 3b).

Finally, we discovered a difference in whole blood in the RNA expression of *THOP1*, which was significantly lower in both treated and non-treated patients with RA, compared to healthy controls (Kruskal-Wallis test *p* = 0.001) and was confirmed in the COMBINE validation cohort (Kruskal-Wallis test *p* = 0.02), although there was only a trend towards a difference between the control group and non-treated patients (Fig. 3c). *THOP1* expression was also negatively correlated to DAS28-C-reactive protein (CRP) score with a *p* value of 0.03 and an adjusted *R* ^2^ of 0.09 in PBMCs from 43 untreated female patients with RA (data not shown). Additionally, we also used RNA-seq data from the COMBINE validation cohort to assess the expression of 349 genes out of 377 reported by Okada et al. Only one gene - *DNase gamma* (*DNASE1L3*) - was DE in this analysis after correction for multiple testing (data not shown).

## Discussion

Our data suggest at least three new candidate genes involved in the development of RA: *ERBB2*, *TP53* and *THOP1*. This finding is based on the integration of previous knowledge from RA association studies, our own RNA-seq expression data and comprehensive pathway analysis with replication in the COMBINE validation cohort.

Initially we hypothesised that genes close to previously found genetic association hits might influence disease-related phenotypes through regulation of RNA expression. This type of association may point to other genes important in RA development, which are part of the same disease-related pathway but do not exhibit a significant change in allelic frequencies defined in conventional GWAS.

The selection of genes proximal to associated loci was based on the criteria previously utilized by Okada et al. [12]. By using this approach we first identified that 11 genes, proximal to validated RA-associated genetic variations, were indeed differentially expressed in whole blood from patients with RA in comparison to healthy controls. Notably, samples could be reasonably grouped into RA and non-RA based on this expression profile alone. This particular gene set, however, did not provide a distinctive clustering between treated and non-treated patients with RA. This circumstance aligns with our intention to avoid genes, displaying heterogeneity of gene expression depending on response to treatment among patients with RA.

Using the IPA service in the second part of the discovery stage, we obtained information about the functional relations between the DE genes from RA-associated loci. As a result, 6 out of 11 input genes were grouped into a single network, where *IFNG* and *TNF* served as connecting hubs. Importantly, this network also contained *HLA-DRB1*. It is notable that shared epitope alleles of this gene are well established as the strongest genetic risk factor of RA. Additionally, TNF was earlier identified as one of the most successful drug targets and currently significant number of patients with RA are receiving anti-TNF treatment [20]. IFNγ is also a well-established contributor to autoimmune reactions during RA course; anti-IFNγ treatment, however, show significant side effects [21]. Thus, discovering that HLA-DRB1, TNF and IFNγ are components in our networks is reassuring in terms of the validity of the integrative approach used in this study.

An important feature of all current treatments for RA is the absence of long lasting post-treatment effect: joint destruction, pain and inflammation reoccur after the cancellation of medication [22]. Based on these observations, we assumed that currently available treatments for RA (including most common methotrexate treatment, anti-TNF treatment and other disease modifying anti-rheumatic drugs, DMARDs) are only palliative and influence the symptoms of inflammation rather than disease-developing pathways. Although we cannot exclude the possibility of DMARDs modifying expression of genes involved in disease pathways, it is tempting to hypothesise that only the physiological changes in RA that are common between treated and non-treated patients in comparison to healthy controls are important for the fundamental mechanisms of the disease. Following this hypothesis, we were prompted to compare non-treated and treated patients versus controls without pooling RA samples into a single group, but rather focusing on common effects in independent patient groups. We used Fisher method for combining p-values as an established approach to distinguish common effects in similar populations with an expected degree of heterogeneity [23, 24]. This approach may be helpful in pointing to gene products that may not be affected by current anti-rheumatic treatments, and has been used previously in expression data analyses [25].

Addressing the expression of the “connector” genes, suggested by the pathway analysis, revealed that some of them were DE in whole blood. Although not previously connected to RA, several of these genes were recently shown to be implicated in autoimmunity (e.g. CARD6 in psoriasis [26], PTGDR in asthma [27], BPI in cystic fibrosis [27]) and immune-related processes [28, 29].

However, with the exception of *HLA-DRB1*, *ERBB2*, *TP53* and *THOP1,* DE was limited to the comparison of healthy individuals to either treated or non-treated RA groups, but not both. This could be potentially attributed to the heterogeneity introduced by treatment. The study design involving both early and established RA was intended to favour genes contributing to disease development, rather then those connected to the acute manifestation of inflammatory symptoms. Therefore, the validation of *ERBB2*, *TP53* and *THOP1* expression in a similarly-structured independent material may point at the importance of these genes in the pathogenesis of RA.

Multiple studies have previously implicated *TP53* in RA pathogenesis, showing that decreased expression on both mRNA and protein level contributes to severe defects in apoptosis, potentially enhancing the severity of autoimmune processes in patients with RA (reviewed in [30, 31]). However, genetic association studies never recognised this gene as associated with RA. Our findings of lower *TP53* expression in patients with RA fall in line with the results of previously published studies.

Our data also indicate lower expression of ERBB2 in whole blood and PBMCs of both treated and non-treated Patients with RA compared to controls. *ERBB2* (HER2/neu) is a receptor tyrosine-protein kinase erbB-2, previously implicated in promoting hyper-proliferative growth in arthritic synovial tissue [32]. Notably, it is known that ERBB2 protein plays an important role in the regulation of the NFkB pathway and, potentially, TNF signalling [33, 34], which are both implicated in RA.

*THOP1* (Thimet Oligopeptidase 1, also known as TOP) was implicated in RA for the first time in this study. Interestingly, *THOP1* has been found to promote rapid degradation of the antigenic peptides, and could affect antigen presentation in vivo [35]. One could speculate that lower expression of *THOP1* observed in whole blood and PBMCs from patients with RA could result in abnormal antigen presentation, which might contribute to the pathogenesis of RA. In this context, it is tempting to investigate a possible functional relationship between *THOP1* and *HLA-DRB1* - the major genetic risk factor for RA.

It is important to mention the limitations of the current study. The discovery cohort is relatively small. Combined with the heterogeneity of gene expression measurements in clinical samples this could lead to insufficient power to detect of some of the genes. Indeed, on testing for DE in the COMBINE validation cohort independently from the discovery cohort, we observed discrepancies that may be indicative of multiple alternative mechanisms leading to differences in regulation of gene expression, different cell composition or more complex timing in this regulation.

Additionally, the RNA in the initial analysis was derived from whole blood, whereas the data from the COMBINE validation cohort is based on RNA from PBMCs. This may explain the discrepant DE results from the two materials on the same gene set, proposed by Okada et al. While useful for verifying and generalizing more consistent signals, this approach does not directly replicate results derived from whole blood. Therefore, it is possible that some of the existing signals could be missed by the current study. The search for pathways underlying RA-associated genes will benefit from larger studies with more stringent replication conditions.

## Conclusion

Integration of RNA-seq data with prior data from association studies and pathway analysis, allowed us to infer new candidate genes and molecular pathways that are potentially involved in RA pathogenesis and could be tested as drug targets. Pathway analysis implies that *ERBB2*, *TP53* and *THOP1* could play a role in a signalling network that could contribute to better understanding of the pathological mechanisms behind RA.

## Additional files

Additional file 1: Table S1. Clinical features of studied groups of patients with RA and healthy controls. **Table S2** The expression and replication status of genes, which were grouped in common functional networks with genes previously associated with RA. (XLSX 55 kb)
Additional file 2: Figure S1. Schematic representation of the study workflow. Out of the initial set of genes in direct proximity to reported RA variants, those DE in our RNA-seq data were assessed using Ingenuity Pathway Analysis software. Interaction molecules suggested by IPA were again compared to the RNA-seq DE results. (TIFF 966 kb)
Additional file 3: Data sheet. RA-associated variants and corresponding proximal genes as defined in the study by Okada et al. (XLS 62 kb)
Additional file 4: Figure S2. Out of the 377 RA-associated genes reported by Okada et al., 22 were differentially expressed in whole blood from either of the RA patient groups, when compared to 12 healthy controls. (TIFF 772 kb)

## Abbreviations

ACPA: anti-citrullinated peptide antibody
ACR: American College of Rheumatology
DAS28: disease activity score (assessment of 28 joints)
DE: differentially expressed
DMARDs: disease-modifying anti-rheumatic drugs
ERBB2: Erb-B2 receptor tyrosine kinase 2
EULAR: European League Against Rheumatism
FDR: false discovery rate
FPKM: fragments per kilobase of transcript per million mapped reads
GWAS: genome-wide association studies
HLA: human leukocyte antigen
IFN: interferon
IPA: Ingenuity Pathway Analysis
NFkB: nuclear factor kappa B
NGS: next-generation sequencing
NSAIDs: non-steroid anti-inflammatory drugs
PBMCs: peripheral blood mononuclear cells
RA: rheumatoid arthritis
SNP: single nucleotide polymorphism
THOP1: thimet oligopeptidase 1
TNF: tumor necrosis factor
TP53: tumor protein P53
