# Supplementary figures and images for: Discovery of new candidate genes for rheumatoid arthritis through integration of genetic association data with expression pathway analysis
Source: Arthritis Res Ther. 2017 Feb 2;19:19. doi: 10.1186/s13075-017-1220-5 (PMC5288892; doi:10.1186/s13075-017-1220-5)

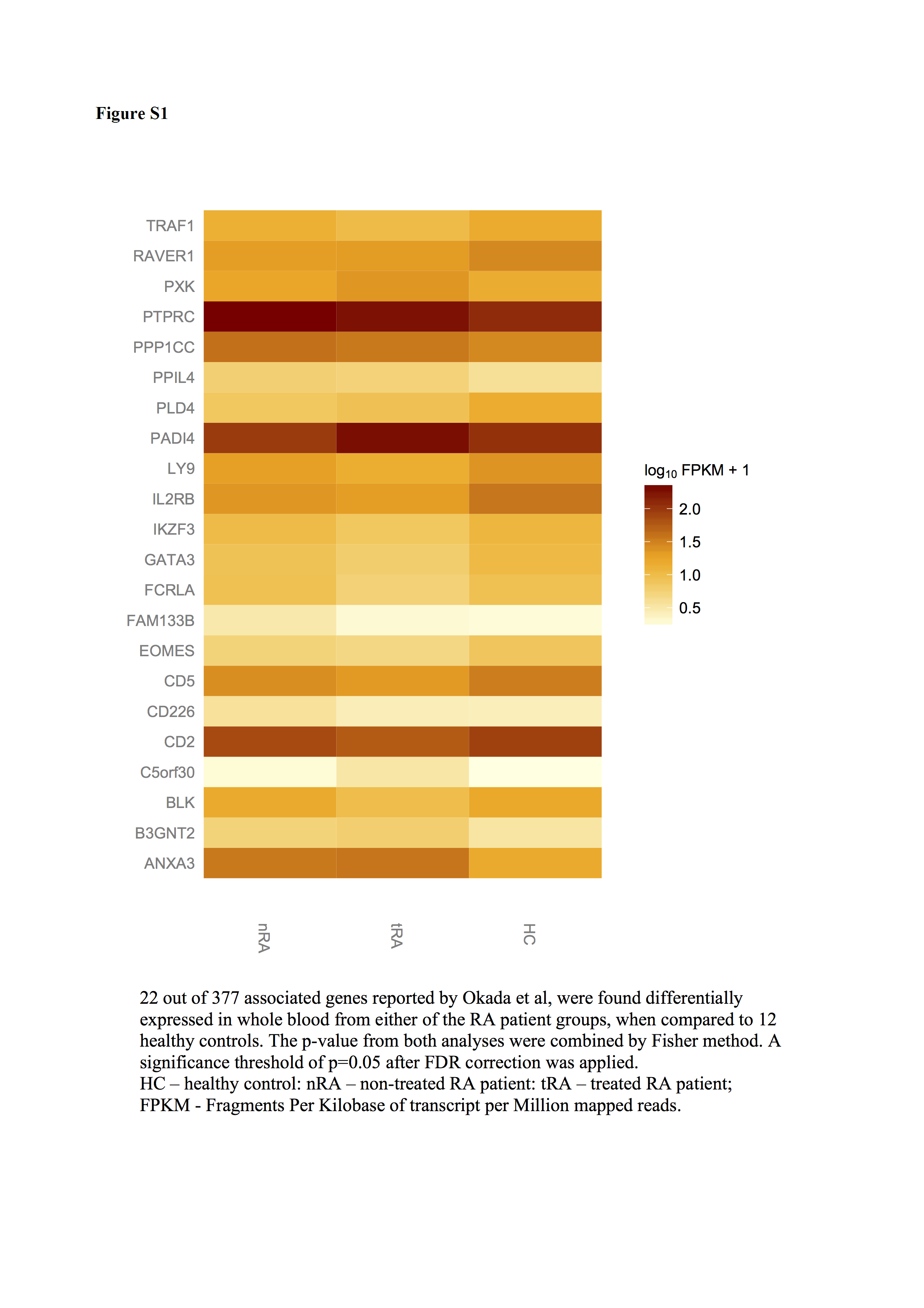

Supplement: Additional file 2: Figure S1. — Schematic representation of the study workflow. Out of the initial set of genes in direct proximity to reported RA variants, those DE in our RNA-seq data were assessed using Ingenuity Pathway Analysis software. Interaction molecules suggested by IPA were again compared to the RNA-seq DE results. (TIFF 966 kb) [file 13075_2017_1220_MOESM2_ESM.tiff]

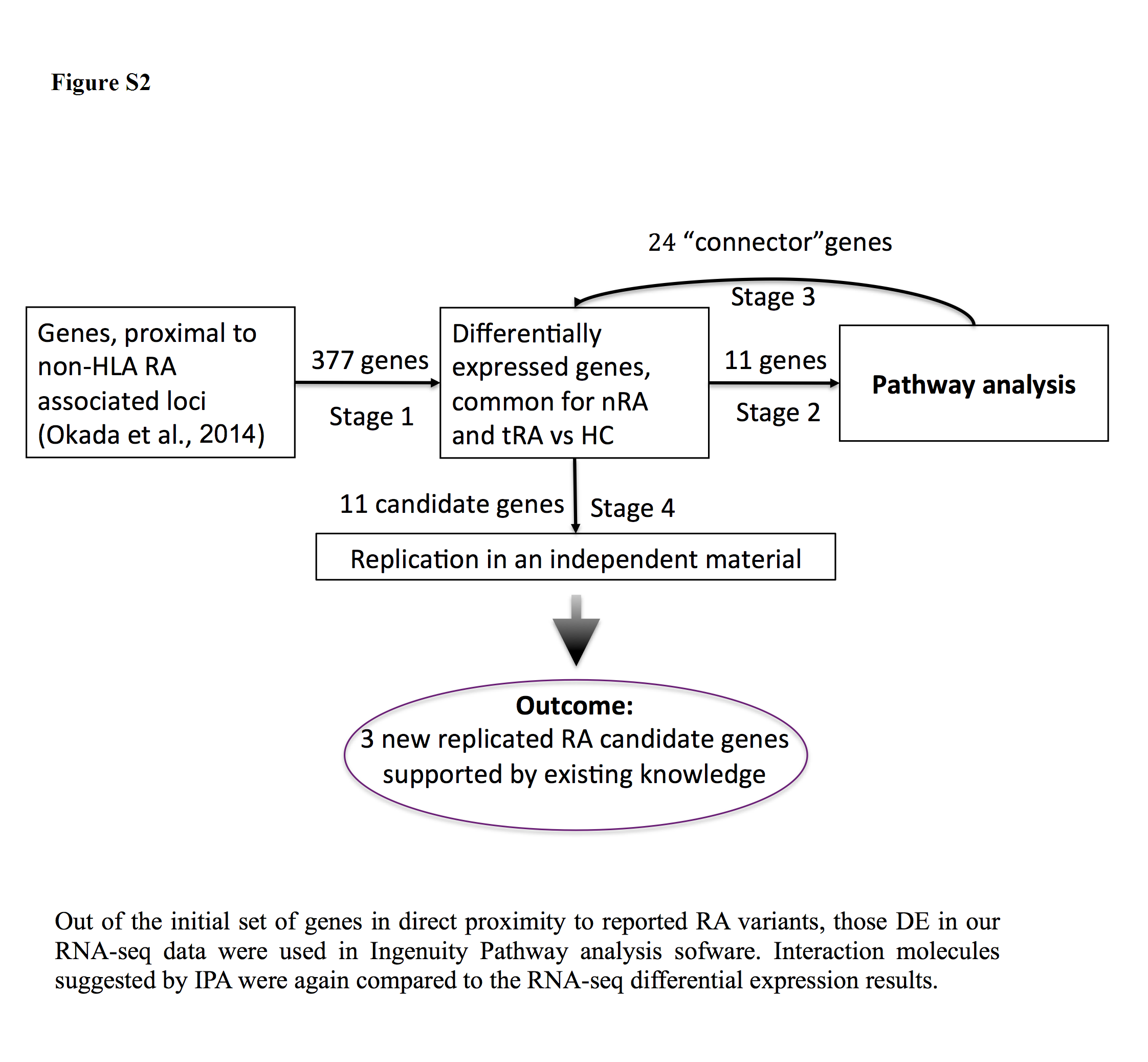

Supplement: Additional file 4: Figure S2. — Out of the 377 RA-associated genes reported by Okada et al., 22 were differentially expressed in whole blood from either of the RA patient groups, when compared to 12 healthy controls. (TIFF 772 kb) [file 13075_2017_1220_MOESM4_ESM.tiff]
